# Setting-Sensitive Conceptualization and Assessment of Quality of Life in Telemedical Care—Study Protocol of the Tele-QoL Project

**DOI:** 10.3390/ijerph181910454

**Published:** 2021-10-05

**Authors:** Klara Greffin, Holger Muehlan, Neeltje van den Berg, Wolfgang Hoffmann, Oliver Ritter, Michael Oeff, Georg Schomerus, Silke Schmidt

**Affiliations:** 1 Department of Psychology, University of Greifswald, 17489 Greifswald, Germany; holger.muehlan@uni-greifswald.de (H.M.); silke.schmidt@uni-greifswald.de (S.S.); 2 Department of Health Care Epidemiology and Community Health, Institute of Community Medicine, University Medicine Greifswald, 17489 Greifswald, Germany; nberg@uni-greifswald.de (N.v.d.B.); wolfgang.hoffmann@uni-greifswald.de (W.H.); 3 Brandenburg Medical School Theodor Fontane, 16816 Neuruppin, Germany; o.ritter@klinikum-brandenburg.de; 4 Department of Cardiology, Brandenburg City Hospital, 14770 Brandenburg an der Havel, Germany; michael@oeff.eu; 5 Department of Psychiatry and Psychotherapy, University Medicine Leipzig, 04103 Leipzig, Germany; Georg.Schomerus@medizin.uni-leipzig.de

**Keywords:** quality of life, telemedicine, patient-reported outcome, questionnaire development, study protocol, chronic disease, mental illness

## Abstract

Quality of life (QoL) is a core patient-reported outcome in healthcare research, alongside primary clinical outcomes. A conceptual, operational, and psychometric elaboration of QoL in the context of TM is needed, because standardized instruments to assess QoL do not sufficiently represent essential aspects of intended outcomes of telemedical applications (TM). The overall aim is to develop an instrument that can adequately capture QoL in TM. For that purpose, an extended working model of QoL will be derived. Subsequently, an instrument will be developed and validated that captures those aspects of QoL that are influenced by TM. The initial exploratory study section includes (a) a systematic literature review, (b) a qualitative survey for concept elicitation, and (c) pre-testings using cognitive debriefings with patients and an expert workshop. The second quantitative section consists of an online expert survey and two patient surveys for piloting and validation of the newly developed instrument. The resulting questionnaire will assess central experiences of patients regarding telemedical applications and its impact on QoL more sensitively. Its use as adjunct instrument will lead to a more appropriate evaluation of TM and contribute to the improvement of care tailored to patients’ individual needs.

## 1. Introduction

Telemedicine (TM) is a vital part of today’s patient care [1,2]. It affects how healthcare services are provided on a structural level, and therewith also influences clinical and patient-reported outcomes (PROs). Quality of life (QoL) is one of the central PROs in the context of TM evaluation studies [3]. However, evidence-based attempts to evaluate the effectiveness of TM applications in improving QoL reveal ambiguous evidence. Although there is some documentation that using TM applications can improve QoL [4], many findings remain inconsistent [5,6,7,8,9,10,11,12]. As such, the reported effects are often not clinically relevant or statistically significant, and they also differ depending on the disease groups studied [13,14]. From a methodological perspective, QoL is frequently assessed with standardized instruments that do not sufficiently represent the most important aspects of the intended outcomes of TM applications. For instance, remote patient monitoring applications are mainly used in heart failure patients to improve distant monitoring of health parameters by medical staff [15]. For the patient, TM use aims to increase patients’ disease-related security [16] and control experience in their personal environment. Those aspects are linked to QoL of patients’, but are not yet assessed within effectiveness studies [17]. Thus, there is a discrepancy between the primary implementation intention and the corresponding evaluation in TM applications.

Furthermore, current assessment-related problems of QoL include that item formulations of PROs are based on very specific disease-related symptoms or experiences. There are challenges in the comparability due to the heterogeneity of TM applications, models of care, and the different target groups [18]. Additionally, effects on QoL are often limited to a selection of specific scales and long-term effects are usually not evaluated [13,14]. Thus, there is a need for a generic patient-centered measurement approach that can capture the expected overall impact of TM [19]. An assessment of QoL in TM derived from such an approach should consequently refer to frequently used TM applications and be based on frequently studied disease groups. Until now, there is no QoL instrument that is sensitive for the TM setting and takes these points into account. As a conclusion, there is a need for a more elaborate conceptual, operational, and hence psychometric foundation of the construct of QoL in the context of TM applications. The Tele-QoL project aims at adapting a general concept of QoL to the TM context. As a next step, an appropriate survey instrument to assess QoL in TM settings will be developed.

## 2. Relevance

An increasing life expectancy and a low birth rate mean the global population is, on average, getting older. This demographic change [20] will increase the demand for TM healthcare solutions. A growing absolute number of older people will lead to increasing age-associated chronic diseases and multi-morbidity [21,22,23]. In order to ensure high-quality healthcare in the future—especially in rural areas—various innovations have been developed in primary and secondary care that have integrated TM applications as a central component [24]. Recently, these TM applications have been the subject of many studies and reviews to investigate or prove their effectiveness [5,6,7,8,9,10,11,12]. While morbidity and clinical indicators have been defined as primary outcome indicators for TM applications in selected disease groups, such as chronic heart failure, QoL has been defined as a primary outcome indicator from patients’ perspective. Reviews [12,25] have shown that it has not yet been possible to document the effect of telemonitoring on QoL, and the findings are inconsistent across specific clinical patient groups and different TM applications [5,6,7,8,9,10,11]. However, the effects of telemonitoring on the daily life of patients, as well as their QoL, well-being, and the subjective experience of control, appear to be considerable: they go beyond the intended health effects and also affect psychosocial and ethical aspects [13,26]. This results in a substantial deficit in considering the patient perspective with regard to the content of patient-reported outcomes in TM studies. The Tele-QoL questionnaire will assess the neglected aspects from a patient’s perspective. Additionally, the project results will have great relevance with regard to different levels:

- *Improvement of TM applications:* A sensitive assessment can result in improvements of TM applications and individual TM care for patients with chronic diseases and mental disorders, making patient-reported and care-relevant information accessible to all professionals. This also includes recommendations on the design of the development, implementation, and evaluation of TM applications to be even better-tailored to patient needs.
- *Improvement of outcome monitoring:* Both the expected increase in numbers of patients and the decrease in the number of primary-care physicians in rural regions require flexible, effective, and evaluated concepts of healthcare provision, particularly to ensure primary care for the population [24]. The expected results of the Tele-QoL project are crucial because they refine the assessment of patient-reported outcome measures (PROMs)/patient-reported experience measures (PREMs) in TM studies. Thus, an enhanced outcome monitoring can be made available in the field of TM care delivery, which takes the patients’ perspective into consideration.
- *Improvement of quality assurance*: The QoL of patients is an essential outcome for therapies. Especially for elderly patients with chronic diseases and psychiatric patients, the focus is often not on full recovery but on disease management, i.e., the limitation of symptoms and circumstantially satisfactory QoL [27,28]. The results of this project are vital, because they improve and extend the recording of PROMs and PREMs in TM studies. This can help to implement more valid and reliable outcome measures in TM-care settings, which, in turn, helps ensure the quality of care.

## 3. Materials and Methods

### 3.1. Ethics

The planned study is committed to the ethical standards of the Declaration of Helsinki. In addition, researchers guarantee to meet relevant legal and ethical requirements as well as all relevant safety regulations. The project was approved by the Ethics Committee at the University Medicine Greifswald (BB 023/18) and the State Medical Association of Brandenburg (AS466 (bB)/2018). Participating patients will be informed about the aims of the project and study procedures in written and oral form. Written informed consent will be obtained from all participants. Although the planned study is not a clinical trial and no specific medical interventions are conducted—apart from those treatments the patients are already receiving independently of the study, as part of their individual treatment plan—there are ethical challenges regarding possible problems caused by potential negative psychological effects arising in the interviews that need to be taken into account. Previous experience with qualitative health-services research among chronically ill patients suggests that such effects are rare and can be avoided if a therapeutically qualified person is available upon request.

### 3.2. Aims of the Study

1. *Gap analysis:* Identification of potential gaps between defined purpose, chosen constructs, and methods of measurement used within TM feasibility and effectiveness studies.
2. *Concept elicitation:* Re-conceptualization of QoL in the context of TM applications.
3. *Instrument development:* Development of an adjunct instrument to assess QoL in TM settings, piloting, and validation study for testing the psychometric performance of the instrument.

### 3.3. Design and Methods

The design includes an explorative study section that consists of a systematic literature review (gap analysis) and a qualitative survey (concept elicitation). A second quantitative approach with structured assessment (instrument development, pre-testing, piloting, and validation of the instrument) will follow.

- *Systematic literature review*: Prior to the empirical investigations, a systematic literature review of existing outcome definitions, criteria of TM applications, and PROM’s/PREM’s from TM studies will be conducted. It aims to identify potential gaps between defined purpose, chosen constructs, and methods of measurement used within TM feasibility and effectiveness studies.
- *Qualitative studies (concept elicitation)*: Interviews and focus groups will be conducted by using semi-structured questionnaires to capture responses and discussions on expectations of, experiences with, and evaluations of TM applications. Additionally, the perceived impact of TM applications on QoL will be explored. While focus groups will be conducted in person, patients and TM professionals will be interviewed in person or via phone. The intensive use of qualitative survey methods is in line with existing recommendations of international PROMs/PREMs research [29].
- *Quantitative studies (testing)*: First, a pretesting of the questionnaire will be conducted, using the cognitive debriefing method “think aloud”. Afterwards, piloting and validation of the instrument for psychometric testing will follow in two independent samples.

### 3.4. Study Participants

#### 3.4.1. Sample Size

*Overall rationale:* The sample of the qualitative and quantitative study part is designed to represent the heterogeneity of TM applications and patient populations to ensure that the results are more generalizable. Therefore, we will include the main groups of TM healthcare professionals and choose patient groups that are contrary with regard to their primary disease (e.g., mental vs. physical chronic disease), but often included in TM studies (e.g., depression vs. heart failure). Additionally, we will examine active and passive TM applications and compare them to standard care. Patients in the active TM application group receive regular phone calls, while patients in the passive TM application group are automatically monitored by a medical device. In essence, we aim to capture a variety of TM experiences from patients with different diseases, of different care spectrums, and from various sociodemographic backgrounds, as well as different TM experts.

*Qualitative studies (concept elicitation)*: The number of focus groups and interviews with *n* = min. 30 is chosen in order to reach content saturation [30,31,32] and is described in Table 1. We aim at the realization of the following:

- Focus groups with a total number of 32 patients to be able to allocate the number of patients from four groups equally (each *n* = 8). The four groups are a combination of patient’s disease (depression/heart failure) and type of care (TM/care as usual).
- Focus groups with a total number of 30 TM professionals.
- Thirty-two single interviews with patients to be able to allocate the number of patients from four groups equally (each *n* = 8). The four groups are a combination of patient’s disease (depression/heart failure) and type of care (TM/care as usual).
- Thirty single interviews with TM professionals.

*Quantitative studies (testing):* Thirty-two patients (cognitive debriefing; see [33]) and at least five experts (expert workshop) will be invited for pretesting. The estimation of the sample size needed for piloting and validation is based on the preconditions of the more complex psychometric procedures such as exploratory and confirmatory factor analysis and differential item functioning. However, the size of the item pool; the communalities of the items; and the number, item sizes, and eigenvalues of the factors are still unknown [34,35]. For the approximation of the necessary sample sizes, we reference simulation studies and reviews [36,37,38]. For piloting, we assume an estimated item size of about 50 ± 10 items, for validation of about 25 ± 10 items. For group comparison with two comparison groups per characteristic, consisting of type of care and disease group, in the final validation step, the approximated ratio of number of cases to items for piloting and validation is approximately equivalent to 4:1 (3:1 to 5:1) and thus corresponds to established practice in the PRO area [36]. Taking reasonable effort and benefit into account, this results in a number of cases of *n* = 200 for the pilot and validation study as a sufficient number for the analyses to be performed.

#### 3.4.2. Recruitment

Recruitment will be implemented by four study nurses in the three recruitment centers of the project’s partners in the German cities of Brandenburg, Greifswald, and Leipzig. The study population will be recruited from patients who are receiving or have received TM care or who are receiving standard treatment for depression or heart failure. The study nurses will contact patients - according to standardized criteria that include type of disease and type of care - with a verbal or written invitation. Patients must suffer from a chronic physical/mental condition or depression/heart failure. Moderate-to-severe impairment of cognitive functions (e.g., comorbid neurological diseases) and non-proficient knowledge of German are exclusion criteria. A research assistant will recruit professionals via email, in person or by phone. Professionals need to be working in the field of TM. All participants had to be at least 18 years old.

#### 3.4.3. Study Assessment and Measures

*Qualitative studies*: The interviews and focus groups will be conducted by using semi-structured interview guides, which will be published within the respective qualitative article.

*Quantitative studies*: Primarily for validation purposes, the following standardized established instruments will be used in addition to the item pool of the newly developed Tele-QoL instrument (see Table 2).

Sociodemographic characteristics will be assessed based on the “Demographic Standards”, a joint recommendation of the Arbeitskreis Deutscher Markt- und Sozialforschungsinstitute e.V. (ADM), the Arbeitsgemeinschaft Sozialwissenschaftlicher Institute e.V. (ASI), and the Federal Statistical Office [39]. We will use a slightly adapted form of a single item for assessing the perceived relative health status from a questionnaire by Renner, Hahn, and Schwarzer (1996) [40]. Moreover, we will phrase questions with regard to disease- or health-related information (e.g., “Do you use telemedicine?”).

Technology commitment will be assessed by using the “Brief measure of technology commitment (TB)” (German original version: [41]). Participants rate their agreement to statements regarding their individual attitudes towards modern technology (e.g., “I am often frightened to fail when dealing with modern technology”) on five response options: 1 = “strongly disagree”, 2 = “disagree”, 3 = “undecided”, 4 = “agree”, and 5 = “strongly agree”. Internal consistency of the subscales “technology acceptance” and “technology competence” was excellent (α = 0.84); for the subscale “technology control”, reliability was good (α = 0.74).

The “Goldman Specific Activity Scale” (original version: [42]) will be used to assess heart failure severity. Participants are asked to rate whether they are able to perform specific daily activities (e.g., “shower without stopping”) and based on their answers classified in four Specific Activity Scale Functional Classes (Class I = least burdened; Class IV = most burdened). It is complemented by the “New York Heart Association Classification” (NYHA; original version: [43]; German version: [44]). The participant must choose the most appropriate statement regarding shortness of breath in daily activities (e.g., “I experience shortness of breath in rest”) in order to be classified in four possible classes (NYHA 1 = least burdened; NYHA 4 = most burdened).

Depressive symptoms will be assessed with the “Patient Health Questionnaire 9 (PHQ-9)” [45]. Participants are asked to rate how often they have been bothered by problems over the last 2 weeks (e.g., “Little interest or pleasure in doing things”), with the following response options: 1 = “not at all”, 2 = “several days”, 3 = “more than half the days”, and 4 = “nearly every day”. Internal reliability (α = 0.89) and test–retest reliability after 48 h (r = 0.84) are excellent.

The new “Tele-QoL” measure will be developed for the assessment of QoL in the context of telemedical care (version A) and standard care (version B) as comparator. Participants evaluate statements regarding their telemedical experiences in the previous four weeks (e.g., “Because of the telemedical treatment, I know how to interpret my symptoms”). The following response options are available: 1 = “strongly disagree”, 2 = “disagree”, 3 = “agree”, and 4 = “strongly agree”. Our study is primarily directed at generating initial evidence for the psychometric performance of the Tele-Qol measure.

With the “SeCu-20” questionnaire (German original version: [46]) participants will be asked to evaluate statements regarding their perceived security in experiences with telemedical care in the last four weeks (e.g., “I can rely on the telemedical application in everyday life”). The response options are 1 = “strongly disagree”, 2 = “disagree”, 3 = “agree”, and 4 = “strongly agree”. The internal reliability of the four scales “technology anxiety”, “perceived security”, “physician–patient relation”, and “perceived autonomy” is good to excellent (α = 0.70–0.89).

Patient satisfaction will be assessed by the “Fragebogen zur Messung der Patientenzufriedenheit (ZUF-8)“ (original version: [47]; German version: [48]). Participants answer questions regarding their general satisfaction with the hospital and the received treatment (e.g., “How satisfied are you with the received treatment generally?”) on four varying response options. Internal reliability is excellent (α = 0.92). Additionally, the general item of the “Youth Health Care Measure (YHC-SUN)” [49] is used to assess the general satisfaction with the treatment (“Have you been satisfied with your healthcare provision in general?”). Response options were 1 = “not satisfied”, 2 = “partly satisfied”, 3 = “satisfied”, 4 = “very satisfied”, and 5 = “extremely satisfied”.

With the “Patient Activation Measure (PAM13-D)” (original version: [50]; German version: [51]), patient activation will be assessed. Participants are asked to evaluate their agreement to statements (e.g., “I know the causes of my symptoms”) on four response options: 1 = “strongly disagree”, 2 = “disagree”, 3 = “agree”, and 4 = “agree strongly”. Internal reliability is excellent (α = 0.84).

To assess body-related self-consciousness, the subscale “private” of the “Body-related Self-Consciousness (KSA)” questionnaire (German original version: [52]) will be used. Participants evaluate their agreement to statements (e.g., “I often can feel my heart beating”) on five response options: 1 = “strongly disagree”, 2 = “disagree”, 3 = “neither agree nor disagree”, 4 = “agree”, and 5 = “agree strongly”.

From the “Body-related Locus of Control (KLC)” questionnaire (German original version: [53,54]) for the assessment of body-related locus of control the subscale “health” will be used. Participants are asked to choose the most appropriate response options for statements (e.g., “Who never falls ill is just lucky”) out of five options: 1 = “strongly disagree”, 2 = “disagree”, 3 = “neither agree nor disagree”, 4 = “agree”, and 5 = “agree strongly”. The internal reliability range is between α = 0.76 und α = 0.79.

The “European Health Literacy Survey (HLS-EU-Q6)” (original version in multiple languages: [55]) will be used to assess health literacy. Participants are asked to evaluate how easy or difficult it is for them to perform different tasks related to health information (e.g., “On a scale from very difficult to very easy, how easy would you say it is to find information on how to manage mental health problems like stress or depression?”). Response options are 1 = “very difficult”, 2 = “fairly difficult”, 3 = “fairly easy”, and 4 = “very easy”. Internal reliability is good (α = 0.80).

Correspondingly, we will implement a newly adapted version of HLS-EU-Q6 for digital healthcare, referred to as D-HLS-EU-Q6. This scale is used to assess digital health literacy by asking patients how easy or difficult they would say it is to perform different tasks regarding digital health information (e.g., “On a scale from very difficult to very easy, how easy would you say it is to find information on how to manage mental health problems like stress or depression with the help of digital health applications?”). The same response options as in the HLS-EU-Q6 are used and preliminary estimation of internal reliability is excellent (α = 0.89).

With the ”WHO-Five Well-Being Index (WHO-5)” [56,57], the QoL of participants with depression will be assessed. Participants are asked how often they felt the described mood in the last two weeks (e.g., “In the last two weeks I felt calm and relaxed”). The available response options are 1 = “all the time”, 2 = “mostly”, 3 = “a little more than half of the time”, 4 = “a little less than half of the time”, 5 = “occasionally”, and 6 = “at no instant”. Internal reliability is excellent (α = 0.92).

The “Minnesota Living with Heart Failure questionnaire (MLHFQ)” will serve for the assessment of the QoL of patients with heart failure [58,59]. Participants rate how often they feel prevented from their wished way of life because of the stated symptoms in the last four weeks. An example question is, “Did your heart failure prevent you from your wished way of life in the last month, whilst you suffered from shortness of breath?” The possible responses are 1 = “very little”; 2, 3, 4, and 5 = “very strong”; and “no” = not applicable. Internal reliability is excellent (α = 0.92; see [60]).

To assess the subjective health status of the participants, the “Veterans RAND 12 Item Health Survey (VR-12)” (original version: [61]; German version: [62]) will be used. Participants are asked about their overall health condition and have the following response options: 1 = “excellent”, 2 = “very good”, 3 = “good”, 4 = “not so good”, and 5 = “bad”. Questions include, how much they feel currently restricted in the stated tasks (response options: 1 = “yes, strongly restricted”, 2 = “yes, a little restricted”, and 3 = “no, not restricted at all”), if they experienced the stated problems at work or in daily activities because of their physical condition and because of their mental health in the last four weeks (response options: 1 = “never”, 2 = “seldom”, 3 = “sometimes”, 4 = “usually”, and 5 = “always”), to which extent pains restricted usual work at home or at work in the last four weeks (response options: 1 = “not at all”, 2 = “a bit”, 3 = “moderate”, 4 = “fairly”, and 5 = “very much”), how often they felt the stated emotions in the last four weeks (response options: 1 = “always”, 2 = “usually”, 3 = “quite often”, 4 = “sometimes”, 5 = “seldom”, and 6 = “never”), and how often physical and mental problems restricted the contact to other people in the last four weeks (response options: 1 = “always”, 2 = “usually”, 3 = “sometimes”, 4 = “seldom”, and 5 = “never”).

The health-related QoL will be assessed with the “European Quality of Life 5 Dimensions (EQ-5D)” (original version: [63,64]). Participants describe their today’s health regarding mobility, taking care of oneself, daily activities, pain/physical afflictions and anxiety/depressiveness on the response options 1 = “no problems”, 2 = “mild problems”, 3 = “moderate problems”, 4 = “big problems”, and 5 = “not able”. Additionally, they evaluate their current health on a visual analogue scale (VAS) from 0 to 100.

The short form of the “World Health Organization Quality of Life (WHOQOL-BREF)” (original version in multiple languages: [65]) will be used to assess the general QoL. Participants evaluate their QoL, life satisfaction, experiences, abilities, satisfaction in different life domains, and negative feelings in the last two weeks on a five-point scale. An example item is “Do you have enough possibilities for leisure activities?” The response phrasing is adapted to the specific type of question. Internal consistency was demonstrated with the following Cronbach’s alpha values: 0.82 in the physical domain, 0.81 in the psychological domain, 0.68 in the social domain, and 0.80 in the environmental domain [65].

### 3.5. Data Evaluation

#### 3.5.1. Data Collection and Management

A data-protection concept will be prepared for the project, and it will be in line with current regulations. This covers information and consent to the study, data collection, data transport, as well as analysis and storage of the data. Moreover, the University of Greifswald will provide a project server and assign access rights among the members of the research team. The project server will be used for the secure storage of project data. Server usage will be in line with currently valid data-protection laws.

Qualitative data will be recorded with a Dictaphone after all participants have given their written informed consent, and related questions were clarified. After the recording, the audio file will be stored on the project server until a transcript of the interview has been made. The audio file will then be deleted, while the transcript remains stored on the server.

Quantitative data will be collected by using questionnaires. The study material will be prepared by the University of Greifswald and subsequently be sent to the recruiting clinical partner institutions. Participants will be asked to return the completed questionnaire material anonymously to the University of Greifswald, using a pre-stamped envelope. After the questionnaires have been received, they will be entered into an Excel spreadsheet and stored on the project server. The original questionnaires will be filed and stored in locked cabinets in rooms to which only limited people with specific clearance have access.

#### 3.5.2. Data Analyses

*Qualitative data*: Qualitative data will be transliterated with the software f4 transcript [66] and subsequently analyzed with MAXQDA software [67]. For the coding of the data material, Mayring’s content analysis approach [68] will be used. The analysis and coding of the transcripts will be made by two persons independently (research assistant, student assistant) and refined iteratively. Possibly deviating codings and contradictory interpretations will be discussed with a supervisor (person in charge of methodology) in a consensual procedure. The analysis will be directed towards identifying all text sequences/units that refer to personal experiences in connection with the application of TM and its impact on QoL. From these contents, categories will iteratively be created, or content will be assigned to existing categories. The resulting category system and the structured contents will be the result of the analyses, which are to be generated from the data input of the qualitative studies. A workshop with experts from the fields of TM applications and QoL research will be conducted for the external validation of the results.

*Quantitative data*: Psychometric analyses on item and scale level, according to classical test theory and item response theory, will be performed after piloting and validation. The piloting will include the selection of an item pool based on the conceptual framework model, the pretesting of the items by cognitive debriefings, the pilot testing of the questionnaire on a sample, and analyses of the descriptive and psychometric performance (e.g., exploratory factor analysis). The validation will include the selection and determination of the final item pool, including scale assignment on the basis of the piloting results and the validation of the measure with an independent sample, as well as analyses of the descriptive and psychometric performance on the item, subscale, and instrument level (e.g., applying confirmatory factor analysis, item response modeling, and differential test functioning).

## 4. Discussion

This research project is expected to generate the following outcomes:

(i) *Patient-related (re-)conceptualization*: The results of the research project address the need for a stronger conceptual elaboration of the construct of QoL in the context of TM applications from the patient’s perspective.
(ii) *Setting-sensitive assessment:* The specific items will assess central expectations and experiences of patients (e.g., perceived security and control beliefs) regarding TM applications more sensitively and can be used as integrated or additional modules of QoL assessments. The instrument allows a more appropriate assessment of the impact of TM on QoL due to increased setting sensitivity.
(iii) *Care-relevant evaluation*: Such a conceptual framework and a corresponding instrument also provide the basis for (re-)evaluating the effectiveness of TM applications through PROMs/PREMs. This potentially allows a re-evaluation of discrepant and inconsistent findings of existing studies on the influence of PROMs/PREMs in general or TM applications on QoL in particular. As a result, evaluations of the influence of TM applications on QoL are more valid and reliable, which will considerably help to improve the funding situation [69]. In addition, the extended assessment of QoL in TM settings will lead to the improvement of these applications—for example, better care tailored to individual cases. Moreover, the results can provide concrete starting points on how this method can be further developed and adapted for other areas. As such, this project contributes to strengthening participatory parts of health-services research.

## 5. Limitations of the Project

The anticipated limitations of the study relate to the inclusion of TM applications. Within this project, those TM applications that are used to complement, but not replace, standard care will be included. Moreover, the landscape of TM is diverse and very dynamic. Consequently, despite aiming for broad applicability, the upcoming results of this study may not be fully generalizable for all future TM applications.

## 6. Conclusions

TM is an important healthcare solution which will continue to become more widespread in the future. The Tele-QoL project aims to add a questionnaire to the field of research, which will enable healthcare professionals, researchers, and stakeholders to assess the impact of TM on QoL more sensitively. This project contributes to advancing telemedical care and to further highlighting the patients’ perspective.

## Figures and Tables

**Table 1:** Sample sizes per study section.

Project Task	Patients	Professionals
Interviews for concept elicitation	*n* = 32 patients in total	*n* = 30 professionals with TM experience
	*n* = 16 patients with chronic diseases (8 each with or without TM care)	
	*n* = 16 patients with mental disorders (8 each with or without TM care)	
Focus groups for concept elicitation	*n* = 32 patients in total	*n* = 30 professionals with TM experience (natural working groups/teams of variable size)
	*n* = 16 patients with chronic diseases (8 each with or without TM care)	
	*n* = 16 patients with mental disorders (8 each with or without TM care)	
Workshop for expert validation of conceptual model		Expert workshop (at least *n* = 5)



Pretesting of item pool	Cognitive debriefings *n* = 32	Online-Survey for Expert-Ratings (at least *n* = 10)
Piloting of preliminary instrument	*n* = 200 total patients	
	*n* = 100 patients with depression (50 each with or without TM care)	
	*n* = 100 patients with heart failure (50 each with or without TM care)	
Validation of final instrument	*n* = 200 total patients	
	*n* = 100 patients with depression (50 each with or without TM care)	
	*n* = 100 patients with heart failure (50 each with or without TM care)	

**Table 2:** Questionnaires and items employed at the different study assessments.

Study Assessments and Measures	Number of Items	Study Time Points		
		Pilot Study	Validation Study (I)	Validation Study (II)
**General information**				
Sociodemographic characteristics	7	X	X	
Perceived relative health status	1	X	X	X
Disease- and health-related information	8	X	X	X
**Psychological instruments**				
Technology commitment (TB)	12		X	
Heart failure severity (Goldman scale & NYHA)	6		X	X
Depressive symptoms (PHQ-9)	10	X	X	X
Quality of life in the context of telemedical care (Tele-QoL-A)	?	X	X	X
Quality of life in the context of standard care (Tele-QoL-B)	?	X	X	X
Perceived security in telemedicine (SeCu-20)	20	X	X	X
Patient satisfaction (ZUF-8)	8	X	X	
Healthcare satisfaction—general item (YHC-SUN)	1		X	
Patient activation (PAM13-D)	13		X	
Body-related self-consciousness—subscale “private” (KSA)	6		X	
Body-related locus of control—subscale “health” (KLC)	5		X	
Health literacy (HLS-6)	6		X	X
Digital health literacy (D-HLS-6)	6		X	X
Disease-specific quality of life—Depression (WHO-5)	5		X	X
Disease-specific quality of life—Heart failure (MLHFQ)	21	X	X	X
Health status (VR-12)	12	X	X	X
Health-related quality of life (EQ-5D)	6		X	
General quality of life (WHOQOL-BREF)	26	X	X	

The selection of questionnaires within a study phase further depends on the group to which the patient belongs (heart failure or depression, with or without telemedical treatment).

## Data Availability

The datasets used and/or analyzed during the current study are available from the corresponding author on reasonable request.
